# Reference Values of Thrombolastometry Parameters in Healthy Term Neonates

**DOI:** 10.3390/children7120259

**Published:** 2020-11-26

**Authors:** Martha Theodoraki, Rozeta Sokou, Serena Valsami, Zoi Iliodromiti, Abraham Pouliakis, Stavroula Parastatidou, Georgia Karavana, Georgios Ioakeimidis, Petroula Georgiadou, Nicoletta Iacovidou, Theodora Boutsikou

**Affiliations:** 1 Neonatal Intensive Care Unit, “Agios Panteleimon” General Hospital of Nikaia, 184 54 Piraeus, Greece; anastasiosmmr@yahoo.gr (M.T.); stavroula.parastatidou@gmail.com (S.P.); gogo_kara@yahoo.gr (G.K.); giorgos.ioakeimidis@gmail.com (G.I.); petroulag@yahoo.gr (P.G.); 2 Hematology Laboratory-Blood Bank, Aretaieio Hospital, National and Kapodistrian University of Athens, 157 72 Athens, Greece; serenavalsami@yahoo.com; 3 Neonatal Department, National and Kapodistrian University of Athens, Aretaieio Hospital, 157 72 Athens, Greece; ziliodromiti@yahoo.gr (Z.I.); niciac58@gmail.com (N.I.); theobtsk@gmail.com (T.B.); 4 2nd Department of Pathology, National and Kapodistrian University of Athens Medical School, University General Hospital Attikon, 124 62 Haidari, Greece; apou1967@gmail.com

**Keywords:** neonatal hemostasis, thromboelastometry, reference range, healthy term neonates, transfusion-guided therapy

## Abstract

Background: Thromboelastometry (ROTEM), as a point of care test, is an attractive tool for rapid evaluation of hemostasis. Currently, no reference ranges exist for all ROTEM assays in neonates, limiting its use in this vulnerable population. The aim of the present study was: (1) to establish reference ranges for standard extrinsically activated (EXTEM), intrinsically activated (INTEM), and fibrinogen polymerization (FIBTEM) ROTEM assays in whole blood samples of healthy term neonates; (2) to determine the impact of gender, delivery mode, and hematocrit on ROTEM parameters. Methods: EXTEM, INTEM, and FIBTEM ROTEM assays were performed simultaneously with complete blood count in 215 healthy term neonates. Results: Reference ranges (2.5th and 97.5th percentiles) were obtained for clotting time (CT), clot formation time (CFT), α-angle, clot firmness at 10 min (A10), maximum clot firmness (MCF), and lysis index at 60 min (LI60, %). Reference ranges for EXTEM were CT 38–78 s, CFT 49–148 s, A10 40–65 mm, and MCF 47–69 mm, LI60 83–98%. For INTEM, CT 134–270 s, CFT 50–142 s, A10 41–63 mm, and MCF 48–67 mm, LI60 85–97%, and finally, for FIBTEM: CT 36–85 s, A10 9–25 mm and MCF 10–26 mm, LI60 92–100%. Hematocrit values were positively correlated with CT, CFT and negatively with A10, MCF values. Conclusion: This study provides, for the first time, reference ranges for ROTEM EXTEM/INTEM/FIBTEM values simultaneously in healthy term neonates. The combined evaluation of ROTEM tests increases its diagnostic accuracy, contributing to the expansion of ROTEM use in the neonatal population.

## 1. Introduction

Viscoelastic methods, such as Thromboelastography (TEG) and rotational Thromboelastometry (ROTEM), evaluate the coagulation process according to the cell-based model of hemostasis, described in 2001 by Hoffman et al. [1], which involves four consecutive phases of clot formation, triggered by tissue factor. These bedside tests estimate all aspects of hemostasis, providing a rapid assessment of clot initiation, clot firmness, thrombin generation, function and number of platelets (PLT), qualitative analysis of the functional fibrinogen component, and fibrin polymerization process. Viscoelastic assays provide in vitro assessment of global coagulation by a numerical and graphical representation of clot formation, clot stabilisation, and clot lysis in a short time and using a small sample of whole blood [2,3].

Conventional tests of coagulation—prothrombin time (PT)—partial thromboplastin time (PTT), international normalized ratio (INR), PLT counts, and fibrinogen—are the most commonly used to assess coagulation. A long turnaround time, inability to diagnose complex coagulation abnormalities, and hyperfibrinolysis are well known flaws of the conventional tests of coagulation, which render them of limited utility in detecting hemorrhage and guiding hemostatic therapy in case of severe bleeding in several clinical settings [4,5]. Additionally, they neither adequately reflect a prothrombotic state, nor do they evaluate the patient’s risk for thrombosis [6].

ROTEM and TEG, as point of care (POC) tests, appear to improve treatment strategies of clotting abnormalities in bleeding patients. They have been increasingly used in diagnosis and goal-directed transfusion therapy [5,7], in adults and paediatric patients, in several clinical situations such as trauma [8], cardiac surgery [9], liver transplantation [10], and postpartum bleeding [7]. Since not only severe hemorrhage, but also blood products transfusions are associated with increased morbidity and mortality, ROTEM guided hemostatic therapy individualized for each patient’s needs with blood products has been shown to be advantageous. It improves patient’s safety and reduces transfusion requirements, complication rates, hospital costs, adverse outcomes, and even mortality [5,7,11]. ROTEM-guided algorithms initially evaluate the INTEM (ellagic acid activated intrinsic pathway) and EXTEM (tissue factor triggered extrinsic pathway) tests. According to the results, the investigation proceeds with FIBTEM, which isolates fibrinogen function (extrinsically activated test with tissue factor and the platelet inhibitor cytochalasin D which blocks platelet activation) and/or APTEM (modified EXTEM assay incorporating aprotinin to stabilize the clot against hyperfibrinolysis) and/or HEPTEM (modified INTEM assay, containing additional heparinase) [7,12].

Neonates, and especially preterms, are the most frequently transfused population in the intensive care units. According to the concept of developmental hemostasis, the coagulation system matures gradually from fetal life throughout adulthood; the levels of most of the coagulation factors are lower in the newborn and they are related to gestational as well as postnatal age. Although standard coagulation tests are often prolonged in the neonatal period compared to that in adult life, reflecting “the hemostatic immaturity”, hemorrhage or thrombosis are rare in the healthy neonate. In case of illness, this “hemostatic balance” is disrupted and the risk of coagulopathy is increased. Disorders of hemostasis in neonates are common incidences in neonatal intensive care units (NICU). Providing a global evaluation of a hemostatic profile and requiring a small amount of blood sample, ROTEM is particularly attractive for use in critically ill newborns. However, limited data exist on its utility in this fragile population, primarily due to the paucity of reference ranges for neonates.

This study aimed at (1) establishing the reference range of ROTEM parameters for EXTEM, INTEM, and FIBTEM assays in healthy term neonates, (2) assessing the impact of delivery mode and gender on the ROTEM parameters, and (3) estimating their correlation with haematocrit values and PLT count.

## 2. Material and Methods

Healthy term neonates born at the General Hospital of Nikaia “Aghios Panteleimon”, Piraeus, Greece over a three-year period (January 2017–February 2020) were the study subjects. The study was concordant with the Declaration of Helsinki and received the approval of the Institutional Review Board and Ethics Committee of the General Hospital of Nikaia-Piraeus (Project identification code: 25.01.2017, 3/1). Parental informed consent was obtained for all neonates included in the study. Gestational age, birth weight, gender, and mode of delivery of the neonates were recorded. Data concerning maternal diseases, medications received during or before the index pregnancy, and complications during pregnancy were also recorded. Healthy term neonates with gestational age ≥37 weeks and appropriate birth weight for gestational age were included in the study. The aim of the present study was to evaluate the ROTEM parameters in healthy term neonates and any neonate hospitalized in the NICU was excluded from the study. Newborns delivered via emergency CS, with a personal or family history of bleeding disorders, with a known or suspected major chromosomal anomaly, septicemia, perinatal blood loss, birth asphyxia, or perinatal stress (defined as neonates with non-reassuring fetal status [13,14] who did not meet the criteria of perinatal asphyxia) were excluded from the study.

Immediately after delivery, 1 mg of vitamin K was administered intramuscularly to all neonates. During the conventional three day hospital stay, all neonates were followed up until discharge. On the second to third day of life, on any occasion of blood testing for various medical conditions (i.e., hyperbilirubinemia, testing for blood group ABO incompatibility, mothers with positive thyroid antibodies, or inadequate medical supervision throughout pregnancy), 900 μL of residual peripheral blood was analyzed on the ROTEM^®^ delta analyzer (Tem Innovations GmbH, Munich, Germany). Three ROTEM tests were performed in single determination (S): EXTEM S, INTEM S, and FIBTEM S. Whole blood had been anticoagulated with 0.109 mol/L (3.2%) trisodium citrate (9:1, *v*/*v* blood anticoagulant). Citrated whole blood (900 μL) was incubated for 2–5 min at 37 °C and was tested 30–60 min after blood collection. The ROTEM tests were performed in single determination using the respective automated pipette programs according to the instructions of the manufacturer. The thromboelastometry technique has been described in detail elsewhere [15].

Blood specimens were carefully evaluated for fibrin clots and any unsuitable specimen was subsequently discarded. Various ROTEM parameters were measured: clotting time (CT, seconds), the time elapsed from the start of measurement until the formation of a clot 2 mm in amplitude; clot formation time (CFT, seconds), the time elapsed from the end of the CT (amplitude of 2 mm) until a clot firmness of 20 mm was achieved; amplitude was recorded at 5 and 10 min (A5, A10); α angle (α °), the angle between the central line (*x*-axis) and the tangent of the ROTEM tracing at the amplitude point of 2 mm, describing the kinetics of clot formation; maximum clot firmness (MCF, mm), the final strength of the clot; lysis index at 30, 45, and 60 min (LI30, LI45, LI60, %), the percentage of remaining clot stability in relation to the MCF following the 30, 45, and 60 min observation period after CT and indicating the speed of fibrinolysis.

In parallel with ROTEM assays, additional blood tests were performed on the study population, which included full blood count, peripheral blood smear, and bilirubin. Complete blood counts were performed on Sysmex XE-2100 analyzer (Roche, Lincolnshire, Illinois, USA). Bilirubin was measured by EXL DIMENSION Analyzer (SIEMENS, Healthcare Diagnostics, Newark, DE, USA). ABO and Rhesus blood group were documented for all neonates under study.

### Statistical Analysis

Data were collected in logistic spread sheets and subsequently, the statistical analysis was performed by SAS-9.4 (SAS Institute Inc. Cary, NC, USA) software. Since normality was not possible to be ensured for all the arithmetic data, the results are presented as median value and the quartile 1 (Q1) to quartile 3 (Q3) range [interquartile range (IQR)]. The categorical data (such as gender) are presented as frequencies with the relevant percentages. The reference ranges were produced as the range between the 2.5 and 97.5 percentiles. Correlations were performed by the Spearman correlation (*r_s_*) coefficient. Comparisons of the ROTEM parameters between or among groups were performed by the Mann Whitney U (MW) test (for comparisons between two groups) or the Kruskal-Wallis (KW) test (for comparisons among more than two groups). All tests were two sided and the significance level of the study was set to *p* < 0.05.

## 3. Results

A total of 215 healthy term neonates with median gestational age of 39 weeks (IQR: 38–40 weeks) and median birth weight of 3300 g (IQR: 3050–3500 g) were included in the study; 52.1% of them were female. Baseline characteristics of the study population are presented in Table 1.

All parameters of standard EXTEM, INTEM, and FIBTEM ROTEM assays are presented as median values and reference ranges (2.5th and 97.5th percentiles) in Table 2.

Statistical analysis showed that there was no impact of gender on the majority of ROTEM parameters (MW test, *p* > 0.05). INTEM LI45 and LI60 were lower in male neonates, with a minor, although statistically significant, difference (median 96% vs. 97% and 93% vs. 94% for males vs. females, MW test: *p* = 0.0429 and 0.0309, respectively). Moreover, weak correlations of gestational age with INTEM LI30, LI45, and LI60 were observed (*r_s_* = 0.14, 0.16, and 0.17 with *p* = 0.0433, 0.0246, and 0.0159, respectively) and similarly, with EXTEM LI30, LI45, and LI60 (*r_s_* = 0.19, 0.14 and 0.15 with *p* = 0.0043, 0.0341, and 0.0278, respectively), while none of the FIBTEM parameters were correlated with gestational age. Birth weight, despite being strongly dependent on gestational age, was only correlated with INTEM LI60 (*r_s_* = 0.16, *p* = 0.0233). Delivery mode (i.e., vaginal delivery vs. Caesarean section) had no influence on any of the ROTEM parameters (MW test, *p* > 0.05 for all comparisons). Regarding maternal disorders, we observed no relation of gestational diabetes or maternal thyroid disease with the ROTEM parameters (MW test, *p* > 0.05).

The correlation of ROTEM variable values with neonatal laboratory blood test results was assessed. A positive correlation was observed between hematocrit values and INTEM CT, CFT and L30, L45 (*r_s_* = 0.20, 0.24, 0.19, and 0.15, respectively, *p* < 0.05) and a negative correlation between hematocrit values and INTEM A5, A10 and alpha angle (*r_s_*= −0.17, −0.15, and −0.25, respectively, *p* < 0.05). In the same arena, regarding EXTEM parameters and hematocrit values, positive correlations were observed only for CT and CFT and a negative correlation was confirmed for alpha angle. Finally, hematocrit values were found to be positively correlated with FIBTEM CT, while negative correlations were observed between hematocrit values and FIBTEM A5 and alpha angle.

Regarding hemoglobin values, a significant correlation was noted with INTEM based measurements (detailed data are presented in Table 3). Hemoglobin was positively correlated with EXTEM CT, CFT and a statistically negative correlation was observed with EXTEM A5, A10, MCF and alpha angle. Finally, hemoglobin values were found to be positively correlated with FIBTEM CT, while negative correlations were observed between hemoglobin values and FIBTEM A5 and alpha angle.

With regards to PLT count, positive correlations with INTEM and EXTEM A5, A10, MCF, and alpha angle and negative correlations with INTEM and EXTEM CFT were observed. Correlations of ROTEM parameters with the blood tests results are reported in Table 3. In the FIBTEM assay, cytochalasin D reagent is used to inhibit the platelet contribution in clot firmness, reflecting the adequacy and functionality of fibrinogen. Consequently, the correlation between platelet count and FIBTEM parameters was not performed. Furthermore, all ROTEM parameters were comparable between neonates with different ABO (KW test: *p* > 0.05 for all comparisons) and Rhesus blood groups (MW test: *p* > 0.05 for all comparisons), while no effect of bilirubinemia on ROTEM parameters was observed (MW test: *p* > 0.05).

## 4. Discussion

We present reference ranges for ROTEM parameters of EXTEM, INTEM, and FIBTEM assays in healthy term neonates. Until recently, reference ranges for these three ROTEM assays, performed simultaneously, have not been described in neonates. Due to scarce data, the usefulness of ROTEM to guide transfusion therapy in this population is limited.

One of the main problems of neonates hospitalized in NICUs is hemostatic disorders. They may become severe and life-threatening; therefore, their diagnosis and management are extremely important for the neonatologist. Hemostasis is a developmental process, constantly evolving from the fetal period until adulthood. Neonates are born with a complex hemostatic deficit, which depends on gestational age, vitamin K levels, and grade of hepatic maturation. There are differences in all coagulation—fibrinolysis system factors between neonates and children or adults. The deficiency of coagulation factors in neonates is functionally counterbalanced by the lower levels of natural inhibitors of hemostasis (Tissue Factor Pathway Inhibitor, antithrombin, protein C, protein S, heparin co-factor II) and the deficits of fibrinolysis components. As a result, healthy term or preterm neonates have no tendency to bleeding or clotting. However, this dynamic equilibrium is deranged in pathologic conditions, including sepsis, asphyxia, injury, etc. In neonates, clinical expression of hemostatic disorders and bleeding manifestation may range from mild (venipuncture-site bleeding) to life threatening. Limited blood volume in neonates and the difficulty to compensate for hypovolemia, which is more prominent in preterm neonates, renders hemorrhage of any grade critical in this patient group. Severe bleeding, with the imminent need of allogeneic blood transfusion, is a complication in NICUs, associated with increased morbidity and mortality. Better understanding of the neonatal hemostatic profile and of the complex functionality of neonatal platelets is also essential.

Conventional coagulation tests, including PT, activated partial thromboplastin time (aPTT), fibrinogen levels, and PLT count, provide data regarding activation of coagulation and consumption of coagulation factors. Nevertheless, their diagnostic capacity is debatable [16], while they present limitations in the provision of hemorrhage and guidance of transfusion therapy in critically ill patients and especially, in neonates [17]. TΕG/ROTEM are diagnostic methods which can uniquely monitor all stages of the hemostatic process. They are conducted in whole blood samples and therefore, analysis encompasses the complex interactions between different cellular blood components and their first results are available within 10 min from the beginning of the test. These viscoelastic methods allow for prompt recognition of various coagulation disorders and accurate treatment guidance. Although ROTEM is utilized as a real time POC test to evaluate the coagulation process and guide evidence based blood product replacement in adults, its use in neonatal transfusion therapy is limited due to the lack of the reference ranges for the basic ROTEM assays.

Most of the studies reporting reference values for ROTEM parameters in neonates have used umbilical cord blood samples [18,19,20]. A few studies have been conducted with neonatal blood samples; however, the population size is small. Additionally, the reference values reported are limited to only one ROTEM test (i.e., EXTEM) [21,22]. Sokou et al. [22] have published reference ranges for EXTEM in term and preterm neonates. Our results for EXTEM assay are within the reference ranges previously established by this group. Oswald et al. [23] have determined reference ranges for EXTEM, FIBTEM, and INTEM assays in different paediatric age groups. One of the study groups included 51 neonates and infants, 0–3 months old, who exhibited accelerated coagulation and strong clot firmness compared to the other groups. Kim et al. [24] aimed to establish reference intervals for the same three ROTEM assays (INTEM, EXTEM, and FIBTEM) in children aged 0–16 years with congenital heart diseases. This study included 119 neonates, who demonstrated a similar hemostatic profile to the population of healthy neonates studied by Oswald et al.

In our study, there was no impact of maternal problems during pregnancy (gestational diabetes, thyroid disease) on the ROTEM neonatal variables. These result are in accordance with previously reported data [22]. Moreover, ROTEM parameters were not influenced by the delivery mode. Schott et al. [25] performed TEG in umbilical blood samples and concluded that the delivery mode did not have an effect on the measured parameters. There were no gender-related differences in the ROTEM variables in our population, except for INTEM LI45 and LI60 being lower in male neonates. Oswald et al. [23] reported similar values of ROTEM parameters between males and females. Among the ROTEM variables tested, LI30, LI45, and LI60 of EXTEM and INTEM assays were positively correlated with gestational age. This finding could be attributed to the lower levels of fibrinolysis inhibitor proteins in earlier gestational weeks [22,26].

Interestingly, a “hypocoagulable” profile, expressed as prolonged CT and CFT in INTEM/EXTEM assays and reduced A5 in INTEM/FIBTEM assays, was observed in neonates with higher hematocrit levels, in keeping with previously published studies [27]. Increased relative red blood cell concentration results in dilution of fibrinogen in whole blood and a subsequent decrease in fibrinogen activity. It has already been highlighted by others that the impact of low hematocrit in patients with anemia should be considered as a possible cause of “hypercoagulability” in ROTEM test results [27,28,29]. As expected, ROTEM parameters were correlated with platelet count, in accordance with reports in literature [3,30].

A limitation of this study is the lack of simultaneous conduction of conventional coagulation tests to correlate with ROTEM parameters. Further large scale trials are required to verify our results.

## 5. Conclusions

To the best of our knowledge, this is the first study to concurrently evaluate three ROTEM assays in a neonatal population, with a relatively large sample size. Considering that the combined evaluation of ROTEM tests increases its diagnostic and therapeutic value, our study results could contribute to the expansion of ROTEM use in the neonatal population. The established reference ranges could possibly support the development of ROTEM-guided transfusion algorithms in neonates.

## Figures and Tables

**Table 1:** Baseline characteristics of the study population.

	Characteristic	Value
Newborns’ data	Gestational age (weeks) Median (Q1–Q3)	39 (38–40)
	Birth weight (g) Median (Q1–Q3)	3300 (3050–3500)
	Gender (males) *N* (%)	103 (47.9%)
Newborns’ laboratory data	Blood group	A (80/37.2%) B (25/11.6%) AB (5/2.3%) O (105/48.8%)
	Rhesus	Positive (195/90.7%) Negative (20/9.3%)
	Hematocrit (%) Median (Q1–Q3)	47.65 (45–51)
	Hemoglobulin (g/dL) Median (Q1–Q3)	16 (15.2–17.1)
	Platelet count (K/μL) Median (Q1–Q3)	285 (240–325)
	Total bilirubin (mg/dL) Median (Q1–Q3)	8 (6–10)
Maternal data	Smoking *N* (%)	20 (9.3%)
	Thrombophilia history	0 (0%)
Pregnancy and delivery	Medication	No medication (184/85.6%) Insulin (1/0.4%) T4 (27/12.6%) T4 and insulin (1/0.4%) Low molecular weight heparin (0/0%) Acetylsalicylic acid (1/0.4%) Propylthiouracil (1/0.4%)
	Mother pregnancy problems *N* (%)	Gestational diabetes (9/4.2%) Autoimmune Thyroiditis (2/0.9%) Gestational Hypothyroidism (24/11.1%) Gestational Hypothyroidism and Diabetes (3/1.4%) Graves (1/0.4%) Hypothyroidism (7/3.2%) Cholestasis of pregnancy (1/0.4%)
	Delivery mode (Cesarean Section) *N* (%)	78 (36.3%)
	Multiple gestation *N* (%)	0 (0%)

**Table 2:** Reference ranges of the ROTEM parameters (*N* = 215 term neonates).

	EXTEM			FIBTEM			INTEM		
Parameter	Median	2.5 Pctl	97.5 Pctl	Median	2.5 Pctl	97.5 Pctl	Median	2.5 Pctl	97.5 Pctl
A5	43	30	56	13	8	22	44	31	55
A10	52	40	65	15	9	25	54	41	63
CFT	86	49	148	-	-	-	76	50	142
CT	52	38	78	48	36	85	191	134	270
LI30	100	98	100	100	97	100	100	97	100
LI45	98	93	100	100	94	100	97	92	99
LI60	95	83	98	100	92	100	94	85	97
MCF	59	47	69	17	10	26	59	48	67
Alpha(α)	73	64	81	74	58	82	75	63	80

Abbreviations: CT, clotting time (seconds); CFT, clot formation time (seconds); A5, A10, clot strength at 5 and 10 min (mm); MCF, maximal clot firmness (mm); LI30, LI45, and LI60, lysis index at 30, 45, and 60 min (%); Pctl, percentile; ROTEM: rotational Thromboelastometry; EXTEM: extrinsically activated; FIBTEM: fibrinogen polymerization; INTEM: intrinsically activated.

**Table 3:** Correlations of ROTEM parameters with blood test results *.

		CT	A5	A10	CFT	MCF	Alpha	LI30	LI45	LI60
**INTEM**	Gestational age (weeks)	0.0103	0.007	0.0289	0.0687	0.0894	−0.0718	0.14062	0.1569	0.17196
		0.8828	0.9165	0.679	0.3241	0.1991	0.3027	**0.0433**	**0.0246**	**0.0159**
	Birth weight (gr)	0.0686	0.03761	0.04179	−0.0067	0.07548	−0.0084	0.07672	0.1311	0.16317
		0.325	0.5896	0.549	0.9238	0.2786	0.9045	0.2718	0.061	**0.0223**
	Hematocrit	0.1956	−0.174	−0.1451	0.23961	−0.0948	−0.2451	0.13712	0.1928	0.15104
		**0.0064**	**0.0155**	**0.0441**	**0.0008**	0.19	**0.0006**	0.0579	**0.0077**	**0.0413**
	Hb (g/dL)	0.2174	−0.2459	−0.2258	0.29104	−0.1638	−0.2855	0.16442	0.2244	0.17762
		**0.0017**	**0.0004**	**0.0011**	**<0.0001**	**0.0187**	**<0.0001**	**0.0185**	**0.0013**	**0.0128**
	PLT (/μL)	−0.0118	0.41007	0.39177	−0.3689	0.3588	0.33725	−0.0806	−0.1195	−0.1096
		0.8668	**<0.0001**	**<0.0001**	**<0.0001**	**<0.0001**	**<0.0001**	0.2504	0.0894	0.1263
	Total bilirubin (mg/dL)	0.0253	0.04715	0.03787	−0.0538	0.0134	0.04437	0.01898	−0.0593	−0.0712
		0.7248	0.5117	0.5982	0.4539	0.8522	0.5369	0.7923	0.4131	0.3368
**EXTEM**	Gestational age (weeks)	0.0514	−0.0186	−0.0003	0.09516	0.01582	−0.0750	0.19417	0.1449	0.15225
		0.4534	0.7866	0.9961	0.1644	0.8176	0.2735	**0.0043**	**0.0341**	**0.0278**
	Birth weight (gr)	0.08697	0.0405	0.0434	−0.0045	0.04138	0.01554	0.03285	0.11182	0.10228
		0.204	0.5548	0.5268	0.9481	0.5462	0.8208	0.6319	0.1028	0.1406
	Hematocrit	0.2285	−0.1366	−0.1174	0.19478	−0.0807	−0.2096	0.1007	0.0966	0.09766
		0.0013	0.0568	0.1023	**0.0064**	0.2618	**0.0033**	0.1613	0.1802	0.1801
	Hb (g/dL)	0.2466	−0.2318	−0.2213	0.25178	−0.1839	−0.2592	0.07342	0.1065	0.11515
		**0.0003**	**0.0007**	**0.0011**	**0.0002**	**0.0071**	**0.0001**	0.2861	0.1221	0.0985
	PLT (/μL)	−0.1346	0.33334	0.32655	−0.3399	0.30471	0.28469	−0.1691	−0.0966	−0.1036
		0.05	**<0.0001**	**<0.0001**	**<0.0001**	**<0.0001**	**<0.0001**	**0.0135**	0.1612	0.1374
	Total bilirubin (mg/dl)	−0.0984	−0.0264	−0.0534	−0.0177	−0.08981	0.00626	−0.08282	−0.1182	−0.06774
		0.1627	0.7085	0.4492	0.8025	0.2026	0.9293	0.2401	0.0939	0.3443
**FIBTEM**	Gestational age (weeks)	0.07063	0.05007	0.06083	NA	0.10829	0.00412	0.01901	0.04735	−0.0875
		0.3167	0.478	0.3886		0.1241	0.9555	0.7878	0.5044	0.2238
	Birth weight (gr)	0.1012	0.03128	0.04169	NA	0.04325	−0.0071	0.02571	0.0336	0.10955
		0.1505	0.6577	0.5548		0.5401	0.9233	0.7157	0.6358	0.1274
	Hematocrit	0.22679	−0.14532	−0.10774	NA	−0.05365	−0.18425	0.06064	0.13475	−0.03144
		**0.0012**	**0.0396**	0.1279		0.4494	**0.0123**	0.3925	0.0577	0.6643
	Hb (g/dL)	0.2515 **0.0003**	−0.1735 **0.0135**	−0.1368 0.0522	NA	−0.0814 0.2492	−0.1893 **0.0099**	0.01676 0.8129	0.1140 0.108	−0.0436 0.5465
	Total bilirubin (mg/dL)	−0.0713	−0.01572	−0.05518	NA	−0.0837	0.15371	0.08055	0.0897	0.06423
		0.3269	0.8291	0.4484		0.2496	**0.0429**	0.268	0.2196	0.3877

Abbreviations: CT, clotting time (seconds); CFT, clot formation time (seconds); A5, A10, clot strength at 5 and 10 min (mm); MCF, maximal clot firmness (mm); LI30, LI45, and LI60, lysis index at 30, 45, and 60 min (%); Hb, hemoglobin; PLT, platelet. * Each cell shows the Spearman correlation coefficient and below the *p*-value. *p*-values in bold indicate statistical significance.
